# Open Reduction and Internal Fixation of a Volar Displaced Salter–Harris III Mallet Fracture in a Pediatric Patient: A Case Report

**DOI:** 10.3390/pediatric17040082

**Published:** 2025-08-06

**Authors:** Alexander Baur, Taylor Anthony, Keith Lustig, Michael L. Lee

**Affiliations:** 1 Research Department, Liberty University, Lynchburg, VA 24502, USA; 2 Department of Orthopaedics, Valley Hospital Medical Center, Las Vegas, NV 89106, USA; 3 Anatomy Department, Liberty University College of Osteopathic Medicine, Lynchburg, VA 24502, USA; 4 Hand Surgery, Desert Orthopaedic Center, Las Vegas, NV 89121, USA

**Keywords:** finger injury, bony mallet, ORIF, pediatric orthopaedics, Salter-Harris fracture, athletic injury

## Abstract

**Introduction:** Finger injuries are common in pediatric patients and typically heal well with conservative management. However, rare fracture patterns involving significant displacement and physeal injury, such as the one described in this case, require specialized surgical intervention to ensure proper healing and prevent long-term complications. **Case Presentation:** A 12-year-old left-hand-dominant female presented with pain, swelling, and deformity at the distal interphalangeal (DIP) joint following hyperextension of the left fifth digit. Initial radiographs revealed a volar displaced intra-articular fracture with physis involvement, confirmed by computed tomography (CT) imaging. Conservative management with closed reduction and splinting failed to achieve adequate alignment. Surgical intervention was performed via a dorsal approach, utilizing ORIF with K-wire fixation to restore joint congruity and ensure anatomic alignment. **Outcomes:** Postoperative follow-up demonstrated satisfactory healing, maintained reduction, and resolution of pain with no complications. The patient regained functional use of the digit with minimal stiffness, and the growth plate remained uninvolved during the recovery period. **Discussion:** This case underscores the importance of advanced imaging, early referral, and tailored surgical intervention for rare mallet fractures involving volar displacement and physeal injury. ORIF provided reliable stabilization and optimal outcomes in this complex case. **Conclusions:** Volar displaced Salter–Harris III fractures of the DIP joint are rare and challenging injuries in pediatric patients. This case highlights the role of ORIF in achieving successful outcomes and emphasizes the importance of precise reduction and stabilization to prevent long-term complications.

## 1. Introduction

Mallet finger injuries are commonly encountered in both adult and pediatric populations, typically resulting from axial loading or hyperflexion of the distal interphalangeal (DIP) joint, leading to disruption of the extensor tendon mechanism [1,2]. These injuries are most often managed conservatively, with splinting being the standard treatment for non-displaced fractures or tendon avulsions [3,4]. However, surgical intervention may be warranted in cases with significant displacement, articular involvement, or when conservative management fails [5,6]. While there is a wealth of literature on dorsal mallet injuries, the volar displacement of an intra-articular fragment, especially in pediatric cases, is exceedingly rare [7].

The involvement of the physis, as in this case, adds another layer of complexity, given the critical need to preserve growth potential and joint integrity [8,9]. Salter–Harris III fractures specifically involve the epiphyseal plate and extend into the articular surface, which can predispose patients to growth disturbances and joint dysfunction if not properly managed [10]. Displacement of the epiphyseal fragment may require advanced imaging with computed tomography (CT) to accurately assess the degree of displacement and plan for surgical correction [11]. The decision to pursue surgical management is based on the extent of the displacement, the involvement of the growth plate, and the risk of long-term complications such as joint stiffness or malalignment [12].

In reviewing the current body of literature, no prior case reports have described a displaced volar Salter–Harris III bony mallet finger. The vast majority of mallet fractures described in pediatric patients involve dorsal avulsion fractures, with only occasional references to the need for surgical intervention [7,8,13,14]. The rarity of this case, where the fracture fragment was volarly displaced, provides a unique opportunity for surgical and clinical learning, particularly in the context of pediatric hand injuries.

By sharing this case, we aim to contribute to the limited body of knowledge regarding surgical management of complex mallet finger injuries, particularly those involving rare fracture patterns such as volar displacement. This case emphasizes the importance of recognizing atypical fracture presentations and highlights the potential for surgical intervention to restore function and prevent long-term complications in pediatric patients. It also demonstrates the utility of CT imaging in preoperative planning and underscores the value of early referral to hand specialists for injuries that deviate from typical patterns.

## 2. Case Presentation

A 12-year-old left-hand-dominant female presented to our orthopedic hand clinic four days after sustaining an injury to her left small finger during a fall. The injury occurred when the patient hyperextended her left fifth digit while catching a slipping cheer bag. She immediately experienced pain, swelling, bruising, and deformity at the distal interphalangeal (DIP) joint.

**Figure 1:**
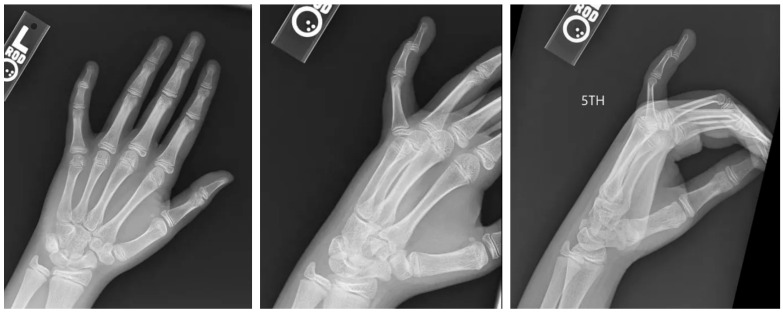
Initial radiographs. AP, oblique, and lateral views of the left hand in a skeletally immature individual demonstrating a dislocated distal phalanx of the small finger.

**Figure 2:**
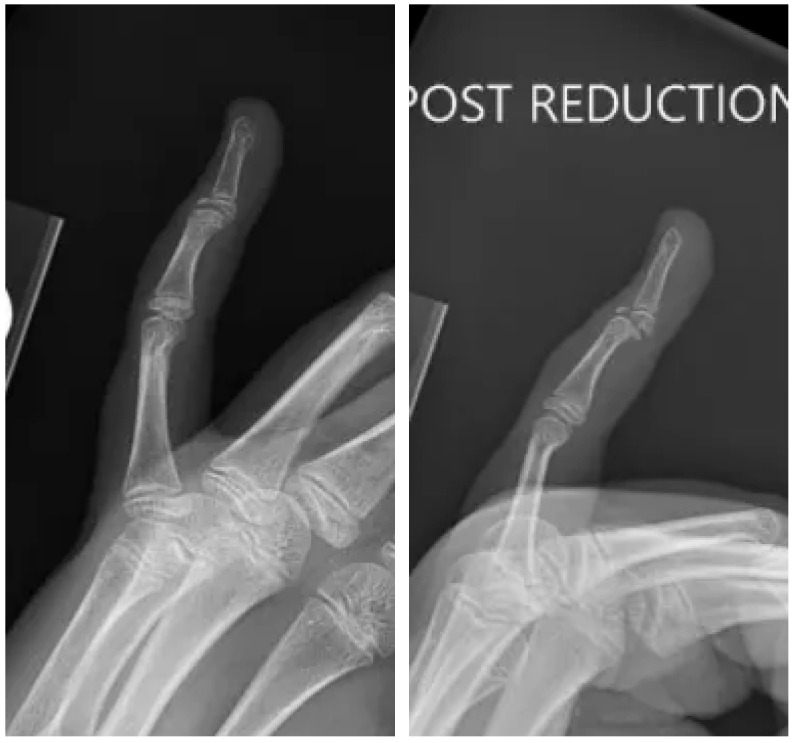
Post-reduction radiographs. Oblique and lateral views of the small finger in a skeletally immature individual demonstrating a reduced distal phalanx with comminuted intra-articular fracture.

The patient initially sought care at an urgent care facility on the same day, where radiographs (Figure 1) revealed an intra-articular avulsion fracture with volar dislocation of the DIP joint. A closed reduction was attempted without anesthesia, followed by immobilization using an AlumaFoam splint. However, post-reduction imaging, as demonstrated in Figure 2, showed residual displacement of the fracture fragment.

**Figure 3:**
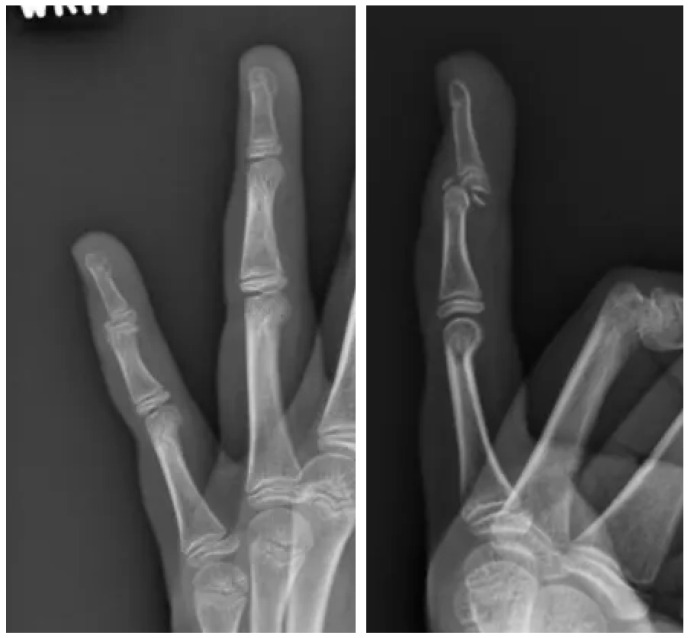
Office visit radiographs. AP and lateral views of the small finger in a skeletally immature individual demonstrating a comminuted intra-articular fracture with volar displacement of the distal phalanx.

At the time of her evaluation in our clinic, the patient reported ongoing pain and difficulty moving her left small finger. On physical examination, there was notable swelling and tenderness over the DIP joint, along with an inability to fully extend the digit. Repeat radiographs (Figure 3) demonstrated a displaced volar fragment involving the physis, consistent with a Salter–Harris type III injury.

To further assess the fracture’s displacement and morphology, a computed tomography (CT) scan was obtained. The CT scan confirmed significant displacement of the volar fragment and its involvement of the growth plate, raising concerns about potential future joint dysfunction and growth disturbance.

Given these findings, surgical intervention was recommended. Six days after the initial injury, the patient underwent open reduction and internal fixation of the fracture through a dorsal approach.

### 2.1. Surgery

The patient was placed in the supine position, and the affected hand was prepped and draped in the usual sterile fashion. Preoperative CT images were reviewed prior to the procedure, which demonstrated a displaced and malrotated volar fragment of the distal interphalangeal (DIP) joint. This imaging helped guide the surgical approach by identifying the fragment’s location and orientation.

**Figure 4:**
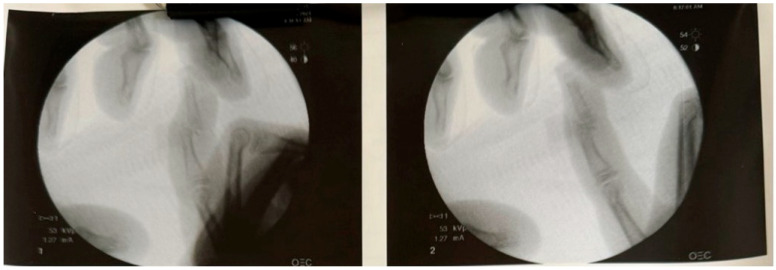

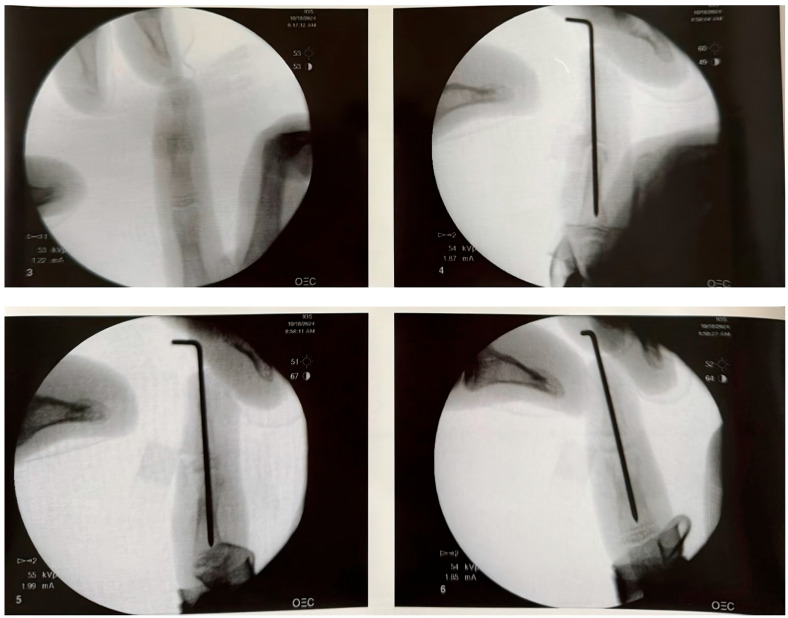
Intra-operative fluoroscopy.

A longitudinal dorsal incision was made over the DIP joint, and careful dissection was carried down to the joint capsule. Upon visualization of the fracture, the preoperative diagnosis of a displaced and malrotated volar fragment was confirmed. However, it became evident that the dorsal incision alone did not provide adequate exposure to the fracture. To improve access, a transverse dorsal incision was made at the level of the DIP joint, allowing for enhanced visualization and mobilization of the volar fragment. The fragment was carefully manipulated and reduced with the assistance of a freer elevator, ensuring its anatomic alignment. Fluoroscopic imaging was utilized intraoperatively to confirm proper reduction (Figure 4).

Once the reduction was deemed satisfactory, a K-wire was inserted through the volar fragment under fluoroscopic guidance to stabilize the fragment and maintain concentric reduction of the joint. Final fluoroscopic imaging confirmed anatomic reduction and stable fixation of the fracture. The surgical site was irrigated thoroughly with copious amounts of sterile saline to ensure a clean wound bed. The incisions were then closed with absorbable sutures used for the skin.

The patient tolerated the procedure well, with no intraoperative complications. Postoperative instructions included immobilization, follow-up imaging to evaluate healing, and clinical assessment for suture removal.

### 2.2. Follow-Up

The patient returned in a follow-up 12 days after surgery wearing a fiberglass 2-finger ulnar gutter splint. She was doing well with mild pain and some stiffness in the small finger. X-rays were obtained and demonstrated concentrically reduced DIP joint transfixed with a K-wire across. The patient planned to continue wearing the splint.

The patient returned 1 month after surgery. X-rays were obtained and demonstrated that the DIP joint remained concentrically reduced with a K-wire across the DIP joint. It was decided to remove the pin in sterile fashion. It was planned to have the patient resume limited activity with a 2-pound lifting restriction for 1 week followed by a 5-pound lifting restriction for 1 week.

At the 6-week follow-up, the patient demonstrated satisfactory progression of healing with no reported complications. Range of motion had improved substantially (0–70 degrees flexion/extension) compared to prior assessments, and clinical evaluation revealed no limitations or concerning findings. The patient was subsequently cleared to resume full activities, including participation in sports.

## 3. Discussion

The presented case of a volar displaced Salter–Harris III fracture dislocation at the distal interphalangeal (DIP) joint in a pediatric patient represents an exceedingly rare and challenging injury. Unlike mallet finger injuries, which involve extensor tendon disruptions or dorsal avulsion fractures, this case was a true fracture dislocation with significant volar displacement of an intra-articular fragment and physeal involvement [15]. Additionally, the mechanism of injury, involving distraction rather than the typical direct blow to the finger, deviated from the traditional mallet finger injury pattern described by Khera et al. [16].

This case underscores an essential but often underappreciated aspect of fracture management: not all reductions are straightforward, and they are not without risk. The initial attempt at closed reduction in this patient, performed without anesthesia, resulted in worsened displacement of the volar fragment. This complication not only increased the complexity of subsequent treatment but also illustrates that reductions, particularly in complex fracture dislocations, must be approached with caution. Miller et al. discussed in their study that volar DIP dislocations may not even need to be reduced [5].

Reduction maneuvers require a precise understanding of the fracture and dislocation mechanics to avoid exacerbating the injury. This experience highlights the importance of careful pre-reduction planning and the role of imaging in guiding decision-making, especially in injuries involving the physis.

Computed tomography (CT) played a pivotal role in this case by providing critical insights into the fracture morphology that were not evident on plain radiographs. The CT scan confirmed significant volar displacement and physeal involvement, clarifying the nature of the injury and allowing for precise surgical planning. Without this detailed imaging, it would have been difficult to appreciate the true extent of the dislocation and the exact orientation of the fracture fragment, which were essential for choosing the appropriate surgical approach.

This case illustrates the value of advanced imaging not only in planning a surgical intervention but also in evaluating cases where initial management fails. CT should be considered a critical tool in assessing complex fracture dislocations, particularly those involving the growth plate, where even subtle malalignments can have long-term implications for joint function and growth.

Prucz et al. also highlights the value of CT when additional views with plain radiographs are not sufficient [17]. Similarly, the review by Sundaram discusses the use of CT and 3D reformatted images for examining the articular surface [18].

Given the significant displacement and involvement of the intraarticular surface, open reduction and internal fixation (ORIF) was the treatment of choice for this injury. Conservative management, while often sufficient for non-displaced or minimally displaced fractures, would have been inadequate in this case due to the severity of the dislocation and the involvement of the growth plate [15].

The surgical approach, using a dorsal incision with enhanced exposure through a transverse incision, allowed for accurate visualization and manipulation of the volar fragment. Due to the extensive injury pattern, the single extensile approach was superior to a dual dorsal and volar approach. Multiple studies on the approach to the DIP joint for arthroplasty have shown positive outcomes for the extensile dorsal approach [19,20]. This approach also minimized the risk of further physeal injury, a critical consideration in pediatric cases. ORIF with K-wire fixation provided stable alignment and restored joint congruity, ensuring optimal conditions for healing.

The patient achieved excellent clinical outcomes, with restored joint congruity, resolution of pain, and minimal residual stiffness. Importantly, the growth plate remained uninvolved during follow-up, reflecting the success of precise surgical intervention. This outcome reinforces the importance of advanced imaging, meticulous surgical planning, and early intervention in complex pediatric injuries. Early referral to specialists with expertise in pediatric hand injuries is essential for optimizing outcomes.

Future studies and case series could further elucidate the optimal management of fracture dislocations in pediatric patients, including the role of advanced imaging and the timing of surgical intervention. Comparative studies evaluating outcomes of various surgical approaches would also contribute to the growing body of knowledge in this area.

## 4. Conclusions

This case highlights the successful use of ORIF in managing a pediatric patient with a volar displaced Salter–Harris III mallet fracture. Prompt surgical intervention allowed for precise reduction and optimal functional outcomes. Early recognition and appropriate treatment of such injuries are essential to prevent complications and preserve hand function in pediatric patients.

## Data Availability

The original contributions presented in this study are included in the article. Further inquiries can be directed to the corresponding author(s).
